# Graves’ Disease and Rheumatoid Arthritis: A Bidirectional Mendelian Randomization Study

**DOI:** 10.3389/fendo.2021.702482

**Published:** 2021-08-17

**Authors:** Dide Wu, Wei Xian, Shubin Hong, Boyuan Liu, Haipeng Xiao, Yanbing Li

**Affiliations:** Department of Endocrinology, The First Affiliated Hospital, Sun Yat-sen University, Guangzhou, China

**Keywords:** rheumatoid arthritis, Mendelian randomization, causal relationship, autoimmune disease (AD), Graves’ disease (GD)

## Abstract

**Background:**

The frequent coexistence of Graves’ disease (GD) and rheumatoid arthritis (RA) has been cited and discussed in observational studies, but it remains a question as to whether there is a causal effect between the two diseases.

**Methods:**

We retrieved genome-wide association study (GWAS) summary data of GD and RA from BioBank Japan (BBJ). Single nucleotide polymorphisms (SNPs) associated with diseases of interest were selected as instrumental variables (IVs) at a genome-wide significance level (*P *< 5.0 × 10^−8^). The random-effects inverse variance weighted method (IVW) was used to combine the causal effect of IVs. The horizontal pleiotropy effect was analyzed by MR-Egger and weighted median method sensitivity test. A leave-one-out analysis was conducted to avoid bias caused by a single SNP. The statistical power of our MR result was calculated according to Brion’s method.

**Results:**

Our study discovered a bidirectional causal effect between GD and RA. The presence of RA may increase the risk of GD by 39% (OR 1.39, 95% CI 1.10–1.75, *P* = 0.007). Similarly, the existence of GD may increase the risk of RA by 30% (OR 1.30, 95% CI 0.94–1.80, *P* = 0.112). Our study provides 100% power to detect the causal effect of RA on GD risk, and vice versa.

**Conclusions:**

We found a bidirectional causal effect between GD and RA in an Asian population. Our study supported the clinical need for screening GD in RA patients, and vice versa. The potential benefit of sound management of RA in GD patients (or GD in RA patients) merits excellent attention. Moreover, novel satisfactory medicine for RA may be applicable to GD and such potential is worthy of further investigation.

## Introduction

Graves’ disease (GD) is an autoimmune disease characterized by the overproduction of thyroid hormones from a diffusely enlarged and overactive thyroid gland. It is the leading cause of thyrotoxicosis and affects approximately 0.5% of males and 3% of females (1). Excess thyroid hormones cause a broad spectrum of symptoms and signs such as palpitations, tremor, agitation, anxiety, weight loss, heat intolerance, and fatigue (2). Patients with severe conditions may have atrial fibrillation, congestive heart failure, hepatomegaly, jaundice, confusion, and even thyroid storm, which is the most extreme clinical expression of thyrotoxicosis (3).

GD seriously affects quality of life and is associated with an increased risk of death (4). The etiology of GD remains unknown but is thought to be multifactorial: i) genetic abnormalities take the major responsibility for GD development, accounting for 75%–80% of the risk (5); ii) epigenetic determinants that act as an on/off switch can regulate gene expression reversibly and temporarily (5); and iii) environmental factors such as iodine intake, smoking, and psychological stress are frequently noticed (4–6). The loss of immunotolerance and the occurrence of stimulating autoantibodies against the thyrotropin receptors are central to GD. Theoretically, the underlying dysregulated immune system may subsequently target other tissues, in conditions that manifest as comorbidities. Previous observational studies have found that approximately 16.7% of patients with GD have another autoimmune disease (7), with rheumatoid arthritis (RA) being the most frequent.

RA is another chronic autoimmune inflammatory disease that primarily influences joints and causes cartilage destruction, bone damage, and systemic comorbidities. It is one of the most prevalent autoimmune diseases, with an incidence of 0.5%–1% worldwide and shows no declining trend (8, 9). As both are autoimmune diseases, GD and RA share similar pathophysiological mechanisms and gene susceptibility. Several studies noticed a high prevalence of RA in GD, and GD in RA has also been documented (7, 10). However, whether there is a causal effect between GD and RA has not yet been reported.

Mendelian randomization (MR) offers a method to investigate whether an observational association between the risk factor and outcome is consistent with a causal effect. It uses genetic variations as instrumental variants (IVs) and relies on equally, randomly, and independently distributed genetic variants during meiosis (11). MR rests on three assumptions: i) the genetic variant is associated with the risk factor, ii) the genetic variant is not associated with confounders, and iii) the genetic variant influences the outcome only through the risk factor (11, 12). Analogous to a randomized control trial, the confounding and reverse causation bias was minimized due to the nature of the study (13). Therefore, our study took advantage of MR and investigated the bidirectional causal effect between GD and RA.

## Materials and Methods

We used a two-sample MR to investigate the causal relationship between GD and RA in the Asian population. Ethical approval and patient consent are not required in our research because they had been obtained in previous studies (14, 15). According to the Strengthening the Reporting of Observational Studies in Epidemiology (STROBE) guideline, our study is reported (16).

### Main Study Population

Single nucleotide polymorphisms (SNPs) associated with GD and RA were obtained from BioBank Japan (BBJ), the largest non-European biobank, which consists of data on 200,000 individuals of East Asian descent (14). Participants who were recruited and enrolled provided their informed consent, and follow-up surveys were performed between 2003 and 2018 (14). Clinical information and biological samples were collected from Japanese patients from 12 medical institutions and population-based controls in Japan, diagnosed with at least one of 47 diseases (14). Among these 47 diseases, GD (in 597 male patients and 1,579 female patients) and RA (in 874 male patients and 3,325 female patients) were the only autoimmune diseases presented (14). Diagnoses of these diseases were based on the diagnoses of physicians made at cooperating hospitals (17). Patients who were not of East Asian descent or who had received a bone marrow transplant were excluded from the BBJ (17). Samples were genotyped by the Illumina HumanOmniExpressExome BeadChip or a combination of the Illumina HumanOmniExpress and HumanExome BeadChips (14). Genome-wide association study (GWAS) was employed by using the Scalable and Accurate Implementation of GEneralized mixed model (SAIGE, version 0.29.4.2; https://github.com/weizhouUMICH/SAIGE) (14, 18). GWAS summary statistics of the 42 diseases are publicly available from the website (JENGER; http://jenger.riken.jp/en/) and the National Bioscience Database Center Human Database (https://humandbs.biosciencedbc.jp/en/) (14).

### Supplemental Study Population

In addition, we obtained previous summary data on RA from GWAS conveyed by Okada et al. in 2014 (4,873 cases and 17,642 controls from East Asian ancestry) for supplementary analysis of the causal relationship between GD and RA (15). The two sets of SNPs associated with RA are independent. Participants were from the GARNET cohort (BBJ, Kyoto University, IORRA) and the Korea cohort (15). Patients with a diagnosis of RA all fulfilled the 1987 criteria of the American College of Rheumatology for RA diagnosis or were diagnosed by professional rheumatologists (15, 19). Since cigarette smoking may be related to GD and RA, we also obtained summary data on smoking initiation from BBJ to ensure that IVs are not associated with cigarette smoking (20).

### Statistical Analysis

**Figure 1:**
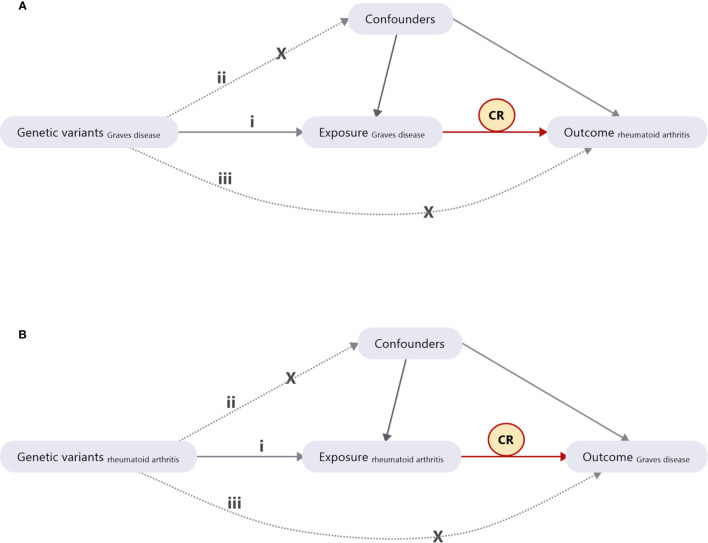
Diagram of Mendelian randomization (MR) study design. **(A)** The causal estimation of Graves’ disease (GD) on rheumatoid arthritis (RA). **(B)** The causal estimation of RA on GD. i) The genetic variants selected as instrumental variables (IVs) should be strongly associated with the risk factor of interest. ii) The genetic variants used as IVs should not be associated with any confounders. iii) The IVs should affect the risk of the outcome merely through the risk factor, not *via* any alternative pathways. CR, causal relationship.

We retrieved GWAS summary data on GD and RA from BBJ along with supplemental summary data on RA. Bidirectional two-sample MR was used to discover the causal relationship between GD and RA. As illustrated in **Figure 1A**, SNPs associated with GD were IVs. Several basic assumptions accompany these IVs: i) they are strongly associated with the phenotype of interest, which in this case is predisposition to GD; ii) they are not associated with confounders of the association between GD and RA; and iii) they are only associated with predisposition to RA through predisposition to GD (12, 21). All of these IVs were associated with GD at a genome-wide significance level (*P *< 5.0 × 10^−8^) to ensure a strong association between IVs and GD. The reverse analysis for the effect of GD on RA is illustrated in **Figure 1B**. When a particular SNP is absent in an outcome dataset, a linkage disequilibrium (LD) proxy SNP can be used (22). We set the threshold of *r* ^2^ at 0.8 to ensure that proxy SNP and target SNP have a strong correlation and that proxy SNP can replace target SNP as IV. SNPs with A/T or G/C alleles are known as palindromic SNPs (22). To ensure that the reference strand is reliable, palindromic SNPs with effect allele frequency between 0.3 and 0.7 were excluded (22). The MR-Egger sensitivity test was applied to analyze the direct pleiotropy effect to ensure that IVs satisfy these assumptions (12). A leave-one-out analysis, which is performed by sequentially dropping one SNP at a time, was conducted to avoid horizontal pleiotropy caused by a single SNP (**Supplementary Figures 1A, B** **)**. The statistical power of our MR result was calculated according to Brion’s method (23).

**Figure 2:**
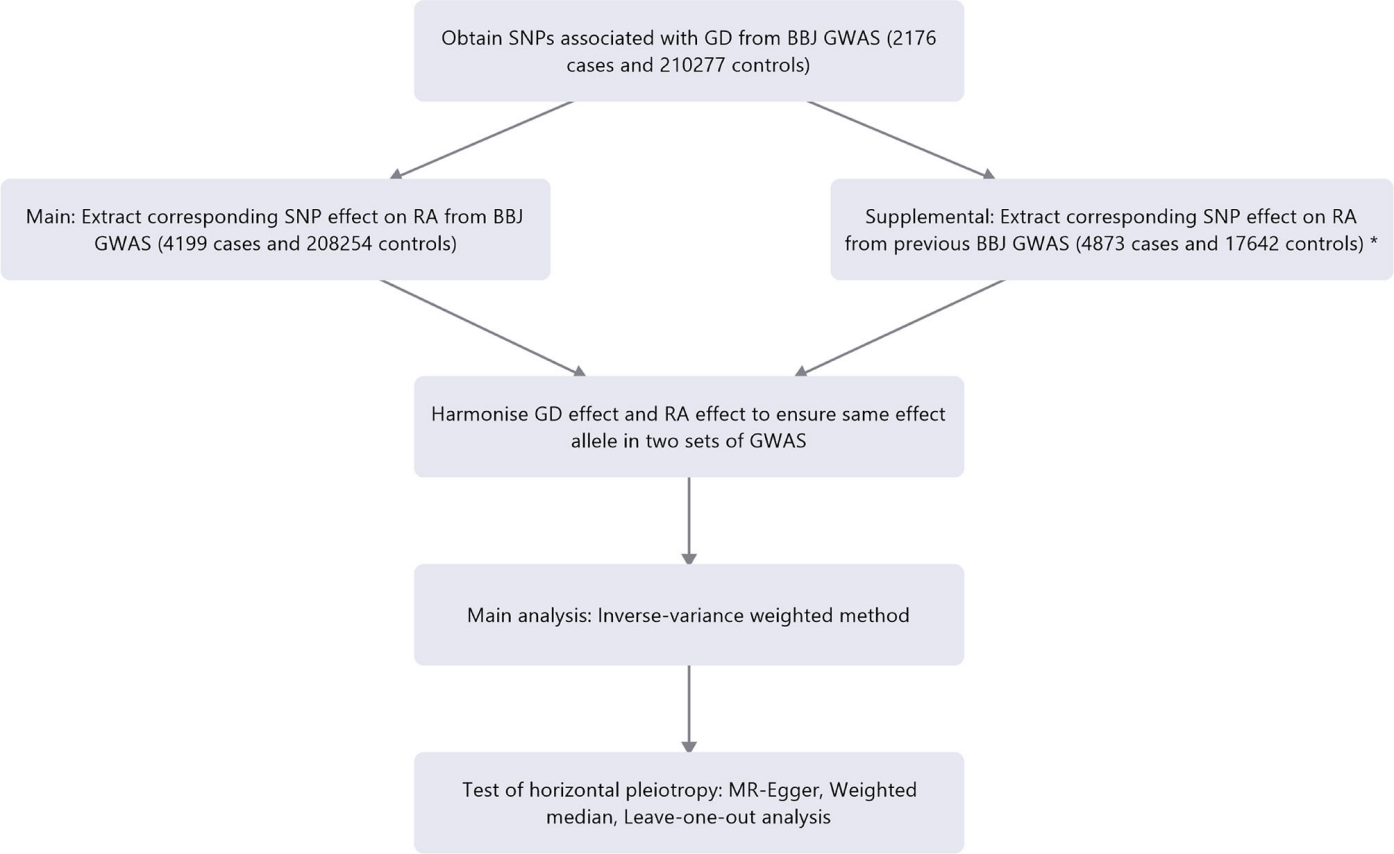
Flowchart of the study process.

Our study conducted various MR methods to confirm the causal relationship between GD and RA. The random-effects inverse variance weighted method (IVW) was used to combine the causal effects of individual SNPs. The MR-Egger regression method and weighted median method were also used to estimate causal relationship. The weighted median method provides robust Mendelian estimates even when 50% of instrument variables are invalid (24). Odds ratio (OR) and 95% confidence interval (CI) were displayed to estimate relative risk caused by the presence of the disease of interest. Results with *P *< 0.05 and power greater than 80% were considered statistically significant. All of the analyses mentioned above were performed using the TwoSampleMR package (version 0.5.5) in R (version 4.0.2). The flowchart to illustrate the process of our study is shown in **Figure 2**. The detailed information of each method used in our study was shown in **Supplementary File 1**.

## Results

From BBJ, of the patients included, 2,176 patients with GD were on average 50.7 ± 15.1 years of age, and 4,199 patients with RA were on average 62.8 ± 12.1 years old. Thirteen SNPs related to GD were extracted to analyze the causal effect of GD on RA. Fifteen percent of the variance was explained for the association between SNPs and GD. Twelve SNPs related to RA were identified as rs3093017 and removed for being palindromic with intermediate allele frequencies. Eleven SNPs were eligible for analysis of the causal effect of RA on GD. Thirteen SNPs related to RA were extracted from a previous study conveyed by BBJ. They explained 4.7% of the variance for the association between selected SNPs and RA. These two sets of SNPs associated with RA are independent. Summary statistics for GD and RA are shown in **Table 1**, and details of SNPs for analysis are shown in **Supplementary Tables 1, 2**. None of the selected SNPs are associated with smoking behavior (**Supplementary Tables 3, 4**).

**Table 1:** Descriptive details of the source of Graves’ disease and rheumatoid arthritis.

Phenotype	Number of SNP	Cases	Controls	Consortium
Graves’ disease	13	2,176	210,277	BBJ
Rheumatoid arthritis	12	4,199	208,254	BBJ
Rheumatoid arthritis*	13	4,873	17,642	BBJ*

The two sets of SNPs associated with RA are independent.

BBJ, BioBank Japan.

*Previous summary data of rheumatoid arthritis from GWAS conveyed by Okada et al.

**Figure 3:**
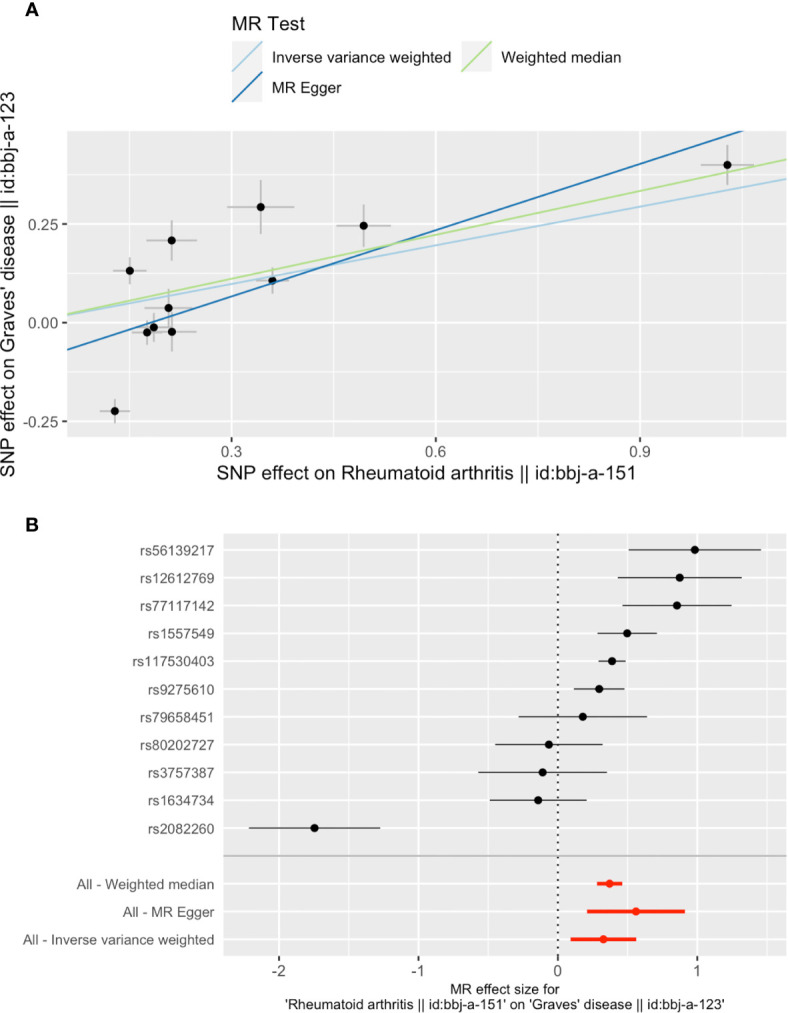
Plots of Mendelian randomization (MR) estimates of the causal relationship between Graves’ disease (GD) and rheumatoid arthritis (RA). The log odds ratio of risk is demonstrated, and three different methods [inverse variance weighted (IVW) approach, MR-Egger, and weighted median] were used. **(A)** The scattered plot of SNPs associated with RA and their risk on GD. **(B)** Forest plot of SNPs associated with RA and their risk on GD. SNPs, single nucleotide polymorphisms.

When setting GD as the outcome, RA was causally associated with GD, as shown in **Table 2** and **Figures 3A, B**. The presence of RA may increase 39% risk of GD (OR 1.39, 95% CI 1.10–1.75, *P* = 0.007). The MR-Egger method (OR 1.75, 95% CI 1.23–2.49, *P* = 0.012) and weighted median method (OR 1.44, 95% CI 1.33–1.58, *P *< 0.001) also indicated consistent results. Directional pleiotropy was not detected when using the MR-Egger method. Sensitivity analysis shows that SNP rs2082260 plays a contradictory role with other SNPs. After removing it, our result still indicated that RA increases the risk for GD (**Supplementary Figure 1A**). Our study provides 100% power to detect the causal effect of RA on GD risk.

**Table 2:** Mendelian randomization estimates of rheumatoid arthritis on Graves’ disease.

Exposure	Method	OR	95% CI	*P-*value	MR-Egger intercept (*P*-value)	Statistical power
Rheumatoid arthritis	Inverse variance weighted	1.39	1.10–1.75	0.007		1
	MR-Egger	1.75	1.23–2.49	0.012	−0.10 (0.131)	
	Weighted median	1.45	1.33–1.58	<0.001		
Rheumatoid arthritis*	Inverse variance weighted	1.36	1.09–1.70	0.007		0.95
	MR-Egger	1.32	0.86–2.03	0.237	0.01 (0.869)	
	Weighted median	1.51	1.36–1.68	<0.001		

The two sets of SNPs associated with RA are independent.

*Previous summary data of rheumatoid arthritis from GWAS conveyed by Okada et al.

**Figure 4:**
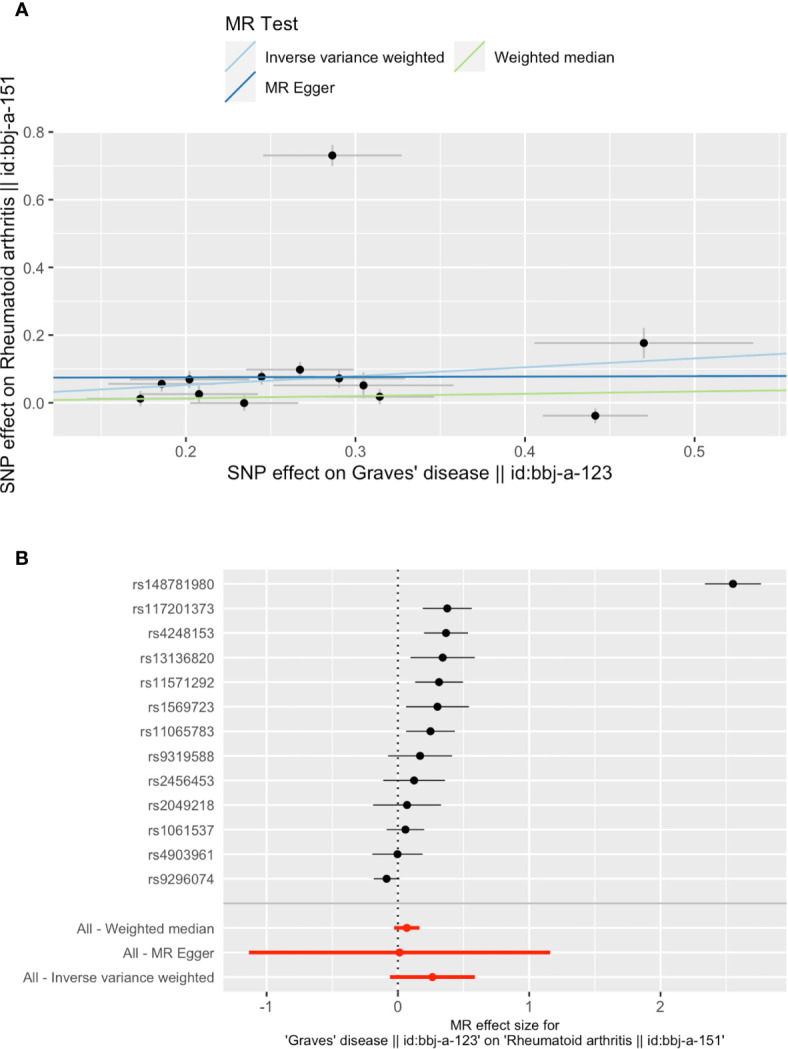
Plots of Mendelian randomization (MR) estimates of the causal relationship between Graves’ disease (GD) and rheumatoid arthritis (RA). The log odds ratio of risk is demonstrated, and three different methods [inverse variance weighted (IVW) approach, MR-Egger, and weighted median] were used. **(A)** The scattered plot of SNPs associated with GD and their risk on RA. **(B)** Forest plot of SNPs associated with GD and their risk on RA (rs148781980 plays a dominant role). SNPs, single nucleotide polymorphisms.

When setting GD as the exposure, the presentation of GD was associated with RA, as shown in **Table 3** and **Figures 4A, B**. The existence of GD may increase the risk of RA by 30% (OR 1.30, 95% CI 0.94–1.80, *P* = 0.112). Results of the weighted median method also support our finding (OR 1.07, 95% CI 0.97–1.18, *P* = 0.197). SNP rs148781980 plays a dominant role in the causal effect of GD on RA, and the result is still consistent when removing this SNP (**Supplementary Figure 1B**). Our study provides 100% power to detect the causal effect of GD on RA. No single SNP significantly biased the causal effect of GD on RA. No significant directional horizontal pleiotropy between RA and GD was presented in the MR‐Egger regression analysis.

**Table 3:** Mendelian randomization estimates of Graves’ disease on rheumatoid arthritis.

Outcome	Method	OR	95% CI	*P*-value	MR-Egger intercept (*P*-value)	Statistical power
Rheumatoid arthritis	Inverse variance weighted	1.30	0.94–1.80	0.112		1
	MR-Egger	1.01	0.32–3.19	0.984	0.07 (0.663)	
	Weighted median	1.07	0.97–1.18	0.067		
Rheumatoid arthritis*	Inverse variance weighted	1.35	0.95–1.94	0.097		1
	MR-Egger	1.06	0.30–3.76	0.927	0.07 (0.702)	
	Weighted median	1.14	1.03–1.26	0.015		

The two sets of SNPs associated with RA are independent.

*Previous summary data of rheumatoid arthritis from GWAS conveyed by Okada et al.

## Discussion

Our study contributed extensive genetic data to the current discussion of whether RA could cause GD, and vice versa, a discussion which, despite arousing attention, leaves open a question that previous observational epidemiological studies have not yet been able to answer. To the best of our knowledge, this is the first study to investigate the bidirectional causal effect between GD and RA using the MR method. We discovered that RA had a positive causal effect on the presence of GD, and vice versa. Our findings suggested that these two common autoimmune diseases may share similar underlying pathophysiological mechanisms. Therefore, novel treatments for RA may also be applicable for GD, and vice versa.

Autoimmune disorders are triggered by the combination of genetic, epigenetic, immunological, and environmental factors, among which genetic predisposition plays a decisive part. Several loci have been identified as contributing to RA and GD susceptibility, including the human leukocyte antigen (HLA) and non-HLA genes.

The HLA complex is located on the short arm of chromosome 6. It is the primary contributor to most autoimmune disorders. The classical theory about the association between HLA locus and autoimmune diseases is due to autoantigen presentation, which affects the selection and activation of autoreactive T cells. Meanwhile, it also possesses a close relationship to autoantibody-mediated autoimmune diseases (25). Despite inconsistencies in various studies, one of the most critical alleles is the HLA DRB1*16:02 that is associated with an increased risk for both GD and RA. The current theory proposes that HLA DRB1*16:02 expressed on B cells may enhance the interaction of B and T cells, causing dysregulated autoreactive B cells and the manufacture of autoantibodies (25).

The protein tyrosine phosphatase nonreceptor type 22 (PTPN22) is the primary non-HLA susceptibility locus for RA, while various polymorphisms showed a close association with GD. This gene is located on chromosome 1p 13.3-13.1 and encodes the lymphoid-specific tyrosine phosphatase (Lyp), which has a high expression level in neutrophils and natural killer cells, and interacts with C-terminal Src kinase to regulate T-cell receptor signaling (26). As of this date, SNP rs2476601 is the most intensively studied autoimmune-associated SNP on PTPN22. It is a gain-of-function mutation, thus increasing the activity of Lyp, leading to inhibition of T-cell signaling and a failure to delete autoreactive T cells (26, 27).

The cluster of differentiation 40 (CD40) gene is a member of the tumor necrosis factor receptor superfamily and closely related to the NF-κB family pathway. The encoded protein is a receptor on antigen-presenting cells of the immune system. Bidirectional interactions between the CD40 and its ligand CD154 (CD40 ligand) are crucial for the generation of both T-cell-dependent humoral immune responses and cytotoxic T-lymphocyte responses. Iscalimab is a nondepleting human anti-CD40 monoclonal antibody that is currently under clinical investigation in several autoimmune diseases, including RA and GD. A marked reduction of thyrotropin receptor antibody was reported in a phase 2 trial (28) and showed potential therapeutic values.

The insulin-like growth factor 1 receptor (IGF-1R) gene is located on chromosome 15 and belongs to the tyrosine kinase receptor family. It is mostly known for being crucial for tumor transformation and survival of malignant cells by activating the PI3K-AKT/PKB pathway and the Ras–MAPK pathway. It also activates the JAK/STAT pathway, which is a critical pathway involved in the inflammatory process and plays an essential role in RA. The elevated level of IGF-IR was documented in both orbital fibroblasts from GD patients and synovial fibroblasts from RA patients, and it has also been found in B and T cells from patients with GD and patients with RA (29, 30). Most recently, teprotumumab, an IGF-1R inhibitor, has shown promising therapeutic value for reducing proptosis in Graves’ ophthalmopathy, which is the most prominent extrathyroidal manifestation of GD. The potential use of teprotumumab in treating RA is worthy of further study. Meanwhile, we are also optimistic about the potential use of JAK inhibitors to treat GD and Graves’ ophthalmopathy.

Besides genetic factors, environmental modulators and epigenetic regulators can influence the predisposition to RA and GD. Given the high comorbidity rate for RA and GD (7), much environmental evidence indicates a close relationship between GD and RA, from which we can envision potential underlying mechanisms. One of the most noticeable shared features is female susceptibility to immune tolerance breakdown (31). DNA methylation, a crucial epigenetic mechanism that can reversibly and temporarily turn genes on or off, plays a crucial role in this complex process (32).

Both GD and RA occur more often in women than in men. A female to male ratio of 6:1 was found in GD, while it is approximately 3:1 in RA (4, 9, 33). The female karyotype includes two X chromosomes, one of which is randomly silenced during embryogenesis through epigenetic mechanisms, especially DNA methylation. A skewed X chromosome inactivation pattern, in which partial genes from the normally inactivated chromosome escape silencing, can result in biallelic expression of some X-linked genes in immune cells. For example, the overexpression of Toll-like receptor 7, which is located on the short arm of the X chromosome and is expressed mainly in plasmacytoid dendritic cells, can lead to excess production of type I interferon and direct links to the initiation and maintenance of autoimmune aggression (34). Moreover, female sex hormones, such as estrogen and progesterone, can enhance the humoral B-cell immune response in normal conditions and contribute to the pathogenesis of GD and RA *via* a broad range of signaling mechanisms. Binding to estrogen receptors, which are widely expressed in immune cells, can promote subsequent gene transcription and directly influence various inflammatory pathways, such as NF-κB signaling, which plays a crucial role in various autoimmune and inflammatory processes (35). Other sex-related factors, including the gut microbiome, susceptibility to infections, and socioeconomic conditions, were also associated with the female predominance in RA and GD, along with other autoimmune diseases (31, 35). Meanwhile, other environmental factors, including diet, smoking, and stress, can also contribute to the initiation and progression of RA and GD *via* epigenetic mechanisms such as histone modification and noncoding RNAs (5, 9).

Since RA and GD have a bidirectional causal effect on each other in addition to the previous evidence from genetic, epigenetic, to environmental levels, good management of one is potentially beneficial to the other. Moreover, they may share ideal drugs if the drug target were showed up in the pathogenesis of the two. Novel effective drugs for RA such as chloroquine, JAK inhibitors, and iscalimab have great potential in the treatment of GD and Graves’ ophthalmopathy. Some of these have been approved in laboratory studies (36) and are more likely to be discovered and proved effective.

Our study has several limitations as well. Firstly, the summary level statistics approach does not allow us to perform analyses stratified by covariates that were adjusted by the original GWAS. Secondly, we did not stratify the causal effects between GD and RA by gender or age. Thirdly, the study population included in the exposure and outcome analyses was of Asian descent. Whether this result could be replicated in Caucasian populations remains a question to be explored.

In conclusion, our study suggested a bidirectional causal association between GD and RA. This has several potential clinical implications for physicians in daily practice. Based on our findings, it is reasonable to consider promoting a routine screening of GD in RA patients, and vice versa. Proper management of RA is essential for downregulating the risk of GD, and vice versa. Meanwhile, satisfactory management of these patients requires the participation of multidisciplinary cooperation. The high efficient multidisciplinary collaboration could play a much more critical role in future clinics and significantly affects the prognoses of patients. Most importantly, the satisfactory therapeutic methods for RA may be applicable in GD as well, such as JAK inhibitors and iscalimab. Our findings provide new evidence for discovering potential drugs for the treatment of GD. The underlying common mechanisms and treatment methods are prone to further development and multidisciplinary teamwork.

## Data Availability Statement

Publicly available datasets were analyzed in this study. These data can be found here: http://jenger.riken.jp/en/ and https://humandbs.biosciencedbc.jp/en/.

## Ethics Statement

Ethical review and approval was not required for the study on human participants in accordance with the local legislation and institutional requirements. The patients/participants provided their written informed consent to participate in this study.

## Author Contributions

DW: conception, design, acquisition, interpretation of data, writing—original draft, and writing—review and editing. WX: data acquisition, analysis, interpretation of data, and writing—original draft. SH: conception and writing—review and editing. BL: analysis, interpretation of data, and writing—review and editing. HX: conception, writing—review and editing, and supervision. YL: conception, design, writing—review and editing, and supervision. All authors contributed to the article and approved the submitted version.

## Funding

This work was supported by the Youth Program of the National Natural Science Foundation of China (No. 81802677) and the Guangzhou Science and Technology Project (No. 201803010057).

## Conflict of Interest

The authors declare that the research was conducted in the absence of any commercial or financial relationships that could be construed as a potential conflict of interest.

## Publisher’s Note

All claims expressed in this article are solely those of the authors and do not necessarily represent those of their affiliated organizations, or those of the publisher, the editors and the reviewers. Any product that may be evaluated in this article, or claim that may be made by its manufacturer, is not guaranteed or endorsed by the publisher.

## Supplementary Material

Supplementary Figure 1 **(A)** The leave-one-out plot of SNPs associated with RA and their risk on GD; **(B)** The leave-one-out plot of SNPs associated with GD and their risk on RA. SNPs, single nucleotide polymorphisms.

## Abbreviations

GD, Graves’ disease; RA, rheumatoid arthritis; MR, Mendelian randomization; IVs, instrumental variables; SNPs, single nucleotide polymorphisms; OR, odds ratio; CI, confidence interval; GWAS, genome-wide association studies; BBJ, BioBank Japan; HLA, human leukocyte antigen; PTPN22, protein tyrosine phosphatase nonreceptor type 22; CD40, cluster of differentiation 40; Lyp, lymphoid-specific tyrosine phosphatase; IGF-1R, insulin-like growth factor 1 receptor.
