# Supplementary figures and images for: Graves’ Disease and Rheumatoid Arthritis: A Bidirectional Mendelian Randomization Study
Source: Front Endocrinol (Lausanne). 2021 Aug 17;12:702482. doi: 10.3389/fendo.2021.702482 (PMC8416061; doi:10.3389/fendo.2021.702482)

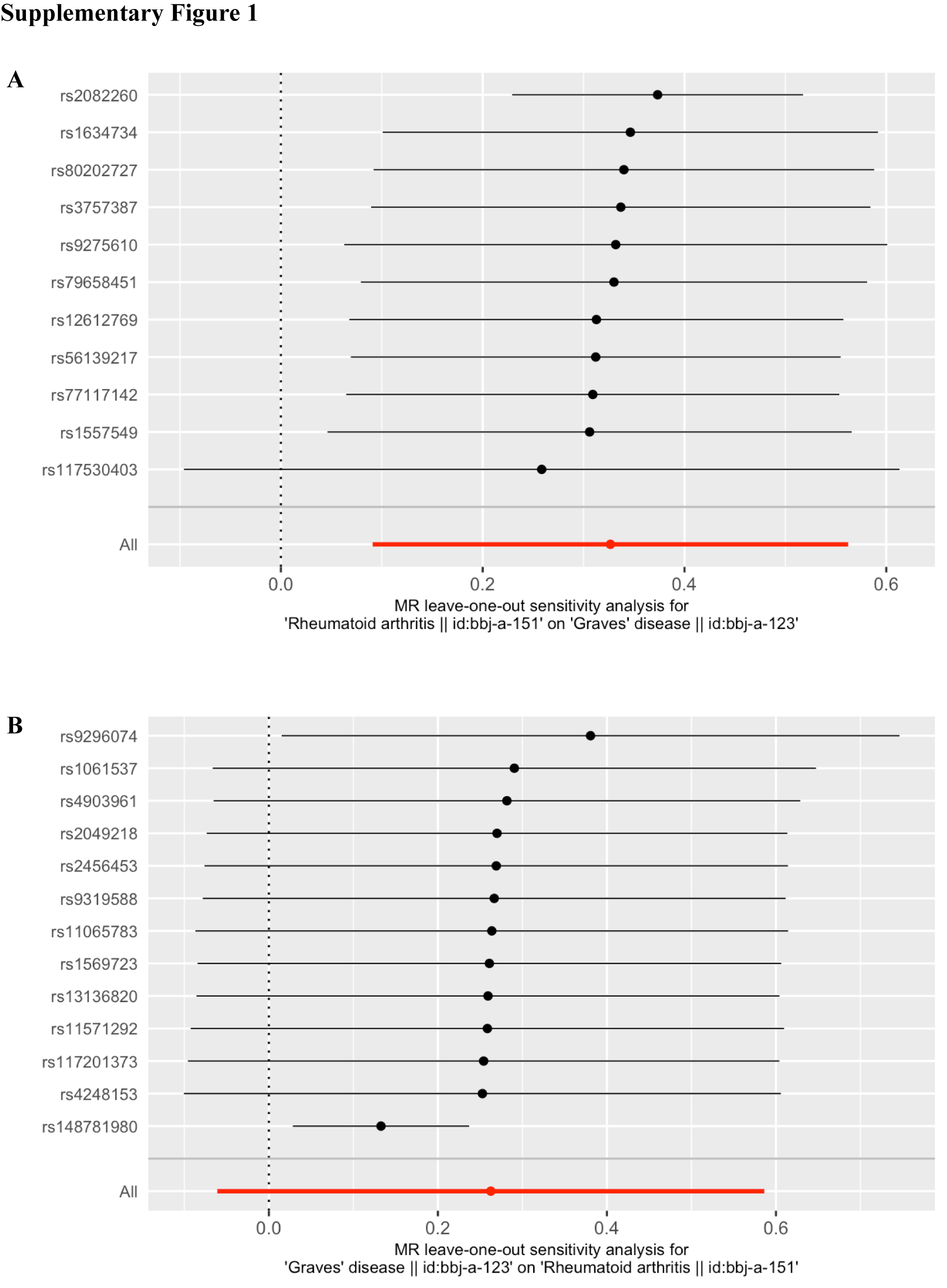

Supplement: Supplementary Figure 1 — (A) The leave-one-out plot of SNPs associated with RA and their risk on GD; (B) The leave-one-out plot of SNPs associated with GD and their risk on RA. SNPs, single nucleotide polymorphisms. [file Image_1.tif]
