# Supplementary material for: Graves’ Disease and Rheumatoid Arthritis: A Bidirectional Mendelian Randomization Study
Source: Front Endocrinol (Lausanne). 2021 Aug 17;12:702482. doi: 10.3389/fendo.2021.702482 (PMC8416061; doi:10.3389/fendo.2021.702482)
Supplement: Supplementary file 2 [file Table_1.docx]

Supplementary Table 1. Details of SNPs associated with rheumatoid arthritis

| Source | Chr | position | SNP | EA | NEA | EAF | β | SE | *P* |
| --- | --- | --- | --- | --- | --- | --- | --- | --- | --- |
| BBJ | 2 | 191953998 | rs12612769 | C | A | 0.3029 | 0.1505 | 0.0247 | 1.06E-09 |
| BBJ | 6 | 34163084 | rs79658451 | C | G | 0.1158 | 0.2074 | 0.0353 | 4.19E-09 |
| BBJ | 6 | 25462110 | rs77117142 | T | C | 0.0565 | -0.3428 | 0.0494 | 3.97E-12 |
| BBJ | 6 | 31307885 | rs1634734 | A | G | 0.4501 | -0.1758 | 0.0225 | 6.27E-15 |
| BBJ | 6 | 32946854 | rs2082260 | T | G | 0.5415 | 0.1285 | 0.0222 | 7.48E-09 |
| BBJ | 6 | 32452282 | rs1557549 | G | A | 0.1287 | -0.4940 | 0.0400 | 5.64E-35 |
| BBJ | 6 | 167541258 | rs3093017 | G | C | 0.5228 | -0.2017 | 0.0230 | 1.58E-18 |
| BBJ | 6 | 32566482 | rs117530403 | G | T | 0.1339 | 1.0283 | 0.0390 | 4.76E-153 |
| BBJ | 6 | 32683750 | rs9275610 | C | T | 0.3283 | -0.3600 | 0.0244 | 2.69E-49 |
| BBJ | 6 | 44249164 | rs80202727 | T | C | 0.2392 | 0.1857 | 0.0265 | 2.47E-12 |
| BBJ | 7 | 128576086 | rs3757387 | C | T | 0.1068 | 0.2123 | 0.0363 | 4.83E-09 |
| BBJ | 10 | 64063077 | rs56139217 | C | T | 0.1138 | 0.2120 | 0.0373 | 1.26E-08 |
| BBJ* | 1 | 17674108 | rs2240339 | T | C | 0.5896 | -0.1833 | 0.0256 | 7.69E-13 |
| BBJ* | 2 | 191943742 | rs11889341 | T | C | 0.3002 | 0.1594 | 0.0274 | 6.28E-09 |
| BBJ* | 6 | 31419823 | rs62395279 | T | C | 0.0149 | 0.6496 | 0.0996 | 6.85E-11 |
| BBJ* | 6 | 32663579 | rs181845116 | G | C | 0.1486 | 0.8299 | 0.0317 | 2.84E-151 |
| BBJ* | 6 | 31588865 | rs114469043 | G | A | 0.0707 | -0.5379 | 0.0585 | 3.52E-20 |
| BBJ* | 6 | 32359460 | rs16870123 | A | G | 0.2738 | 0.4278 | 0.0298 | 1.01E-46 |
| BBJ* | 6 | 32378132 | rs28515648 | A | G | 0.2589 | 0.5997 | 0.0336 | 3.87E-71 |
| BBJ* | 6 | 31395113 | rs144538425 | A | T | 0.1271 | 0.4815 | 0.0390 | 5.21E-35 |
| BBJ* | 6 | 44242789 | rs190669824 | G | A | 0.2162 | 0.2154 | 0.0297 | 4.47E-13 |
| BBJ* | 6 | 167538897 | rs10946216 | C | T | 0.5145 | -0.2430 | 0.0260 | 8.53E-21 |
| BBJ* | 6 | 33799862 | rs4279424 | C | T | 0.2674 | -0.2193 | 0.0319 | 6.30E-12 |
| BBJ* | 6 | 138223489 | rs9494892 | T | G | 0.0682 | 0.2981 | 0.0465 | 1.41E-10 |
| BBJ* | 10 | 63785089 | rs10821944 | T | G | 0.6427 | -0.1609 | 0.0261 | 6.92E-10 |
| BBJ* | 18 | 12773338 | rs2847266 | T | C | 0.5172 | -0.2095 | 0.0330 | 2.17E-10 |

Abbreviations: Chr: Chromosome, SNP: single nucleotide polymorphism, EA: effect allele, NEA: non-effect allele, EAF: effect allele frequency, SE: standard error.

Supplementary Table 2. Details of SNPs associated with Graves’ disease

| Source | Chr | position | SNP | EA | NEA | EAF | β | SE | *P* |
| --- | --- | --- | --- | --- | --- | --- | --- | --- | --- |
| BBJ | 1 | 160419940 | rs117201373 | G | A | 0.0653 | 0.4701 | 0.0646 | 3.29E-13 |
| BBJ | 2 | 204720139 | rs11571292 | A | G | 0.6136 | 0.2447 | 0.0317 | 1.10E-14 |
| BBJ | 3 | 188122978 | rs2049218 | T | C | 0.3887 | -0.1731 | 0.0317 | 4.89E-08 |
| BBJ | 4 | 40307564 | rs13136820 | T | C | 0.7193 | -0.2021 | 0.0351 | 8.40E-09 |
| BBJ | 6 | 29937795 | rs1061537 | A | G | 0.6236 | -0.3143 | 0.0321 | 1.10E-22 |
| BBJ | 6 | 32667577 | rs148781980 | G | A | 0.1773 | 0.2863 | 0.0408 | 2.32E-12 |
| BBJ | 6 | 33042598 | rs9296074 | G | A | 0.4689 | 0.4415 | 0.031 | 4.91E-46 |
| BBJ | 6 | 31002527 | rs4248153 | G | A | 0.6205 | -0.2673 | 0.0318 | 3.91E-17 |
| BBJ | 8 | 128201359 | rs2456453 | T | C | 0.3406 | -0.2077 | 0.0348 | 2.35E-09 |
| BBJ | 12 | 111396249 | rs11065783 | G | A | 0.2695 | 0.2904 | 0.0388 | 7.23E-14 |
| BBJ | 14 | 81462649 | rs4903961 | G | C | 0.6134 | 0.2344 | 0.0319 | 1.96E-13 |
| BBJ | 16 | 30930983 | rs9319588 | T | C | 0.9055 | -0.3047 | 0.0531 | 9.63E-09 |
| BBJ | 20 | 44742064 | rs1569723 | A | C | 0.6152 | 0.1857 | 0.0316 | 4.06E-09 |

Abbreviations: Chr: Chromosome, SNP: single nucleotide polymorphism, EA: effect allele, NEA: non-effect allele, EAF: effect allele frequency, SE: standard error.

Supplementary Table 3. Details of SNPs associated with rheumatoid arthritis and association with smoking initiation

| SNP | Chr | Position | EA | NEA | EAF | β | SE | *P* |
| --- | --- | --- | --- | --- | --- | --- | --- | --- |
| rs12612769 | 2 | 191953998 | C | A | 0.2980 | -0.0003 | 0.0016 | 0.8515 |
| rs80202727 | 6 | 44249164 | T | C | 0.2411 | -0.0004 | 0.0017 | 0.8118 |
| rs77117142 | 6 | 25462110 | T | C | 0.0662 | 0.0054 | 0.0030 | 0.0810 |
| rs79658451 | 6 | 34163084 | C | G | 0.1221 | 0.0037 | 0.0022 | 0.0983 |
| rs2082260 | 6 | 32946854 | T | G | 0.4747 | 0.0003 | 0.0015 | 0.8317 |
| rs3093017 | 6 | 167541258 | G | C | 0.5153 | 0.0001 | 0.0015 | 0.9604 |
| rs9275610 | 6 | 32683750 | C | T | 0.3274 | 0.0017 | 0.0015 | 0.2547 |
| rs1634734 | 6 | 31316760 | A | G | 0.4223 | 0.0013 | 0.0015 | 0.3947 |
| rs3757387 | 7 | 128576086 | C | T | 0.1053 | -0.0023 | 0.0023 | 0.3348 |
| rs56139217 | 10 | 64063077 | C | T | 0.1052 | -0.0008 | 0.0024 | 0.7425 |
| rs2240339 | 1 | 17674108 | T | C | 0.5885 | -0.0011 | 0.0015 | 0.4644 |
| rs11889341 | 2 | 191943742 | T | C | 0.3053 | -0.0007 | 0.0016 | 0.6433 |
| rs114469043 | 6 | 31588865 | G | A | 0.0697 | 0.0063 | 0.0028 | 0.0265 |
| rs9494892 | 6 | 138223489 | T | G | 0.0713 | 0.0027 | 0.0028 | 0.3448 |
| rs16870123 | 6 | 32359460 | A | G | 0.3055 | -0.0021 | 0.0016 | 0.1843 |
| rs181845116 | 6 | 32663579 | G | C | 0.1579 | -0.0045 | 0.0021 | 0.0306 |
| rs10946216 | 6 | 167538897 | C | T | 0.5137 | 0.0001 | 0.0015 | 0.9505 |
| rs190669824 | 6 | 44242789 | G | A | 0.2233 | 0.0007 | 0.0017 | 0.6731 |
| rs4279424 | 6 | 33800297 | C | T | 0.2118 | -0.0018 | 0.002 | 0.3747 |
| rs10821944 | 10 | 63785089 | T | G | 0.6345 | 0.0031 | 0.0015 | 0.0389 |
| rs2847266 | 18 | 12773338 | T | C | 0.5379 | 0.0006 | 0.0014 | 0.6731 |

Abbreviations: Chr: Chromosome, SNP: single nucleotide polymorphism, EA: effect allele, NEA: non-effect allele, EAF: effect allele frequency, SE: standard error.

Supplementary Table 4. Details of SNPs associated with Graves’ disease and association with smoking initiation

| SNP | Chr | Position | EA | NEA | EAF | β | SE | *P* |
| --- | --- | --- | --- | --- | --- | --- | --- | --- |
| rs117201373 | 1 | 160419940 | G | A | 0.0652 | 0.006 | 0.0029 | 0.0399 |
| rs11571292 | 2 | 204720139 | A | G | 0.6135 | -0.0008 | 0.0015 | 0.5937 |
| rs2049218 | 3 | 188122978 | T | C | 0.3951 | -0.002 | 0.0015 | 0.1843 |
| rs13136820 | 4 | 40307564 | T | C | 0.7161 | 0.0013 | 0.0016 | 0.4146 |
| rs4248153 | 6 | 31002527 | G | A | 0.622 | 0.002 | 0.0015 | 0.1843 |
| rs9296074 | 6 | 33042598 | G | A | 0.4663 | -0.0013 | 0.0014 | 0.3747 |
| rs1061537 | 6 | 29937795 | A | G | 0.6296 | 0.0007 | 0.0015 | 0.6235 |
| rs148781980 | 6 | 32667964 | G | A | 0.1678 | -0.0042 | 0.002 | 0.0358 |
| rs2456453 | 8 | 128201359 | T | C | 0.3415 | 0.0039 | 0.0017 | 0.0192 |
| rs4903961 | 14 | 81462649 | G | C | 0.6133 | -0.0016 | 0.0015 | 0.2747 |
| rs9319588 | 16 | 30930983 | T | C | 0.9057 | -0.0024 | 0.0025 | 0.3348 |
| rs1569723 | 20 | 44742064 | A | C | 0.6178 | -0.0027 | 0.0015 | 0.0677 |

Abbreviations: Chr: Chromosome, SNP: single nucleotide polymorphism, EA: effect allele, NEA: non-effect allele, EAF: effect allele frequency, SE: standard error.
