# Supplementary material for: Graves’ Disease and Rheumatoid Arthritis: A Bidirectional Mendelian Randomization Study
Source: Front Endocrinol (Lausanne). 2021 Aug 17;12:702482. doi: 10.3389/fendo.2021.702482 (PMC8416061; doi:10.3389/fendo.2021.702482)
Supplement: Supplementary file 3 [file DataSheet_1.docx]

Supplementary File 1. Detailed illumination for the process of each MR method.

Mendelian randomization(MR) uses genetic variants to infer the causal relationship between risk factors and outcomes, mimicking randomized clinical trials. (1) Suppose we have a single nucleotide polymorphism (SNP) that is known to influence some phenotype (i.e., robustly associated with the phenotype). According to Mendel’s laws of inheritance and the fixed nature of germline genotypes, the alleles an individual receives at this SNP are expected to be random with respect to potential confounders and causally upstream of the exposure (1).

In MR analysis, SNPs robustly associated with risk factors can be used to explore the unconfounded causal effect of that risk factor on an outcome (2). These eligible genetic variants are called instrumental variables (IVs) (2). There are three fundamental assumptions on these IVs: (i) the genetic variant is associated with the risk factor. (ii) the genetic variant is not associated with confounders; and (iii) the genetic variant influences the outcome only through the risk factor. (3, 4)

When concerning the first assumption, the aim is to select genetic variants that are robustly associated with the risk factor of interest. The statistical strength of the genetic instrument-risk factor association if ‘weak’ can result in weak instrument bias. (as demonstrated in <https://mr-dictionary.mrcieu.ac.uk/term/relevance/>). Our study extracts SNPs associated with GD/RA with *P* < 5.0 × 10-8 to ensure a strong association between SNPs and GD/RA and avoid weak instrument bias. When a particular SNP is absent in an outcome dataset, a linkage disequilibrium (LD) proxy SNP can be used (1). We set the threshold of *r^2^* at 0.8 to ensure that proxy SNP and target SNP have a strong correlation and that proxy SNP can replace target SNP as IV. SNPs with A/T or G/C alleles are known as palindromic SNPs because their alleles are represented by the same pair of letters on the forward and reverse strands, which can introduce ambiguity into the identity of the effect allele in the exposure and outcome GWASs (as demonstrated in <https://mr-dictionary.mrcieu.ac.uk/term/palindrome/>). So we exclude palindromic SNPs with effect allele frequency between 0.3 and 0.7 to ensure that the reference strand is reliable. The random-effects inverse variance weighted method (IVW) was used to combine Wald ratios (the causal estimate obtained for a single genetic variant by dividing its gene-outcome association by its gene-exposure association of individual SNPs), effectively treating each SNP as a valid natural experiment (with zero intercepts and a slope parameter)(1).

The second and third assumption needs evaluation of horizontal pleiotropy. Horizontal pleiotropy refers to the effects of the SNP on the outcome not mediated by the exposure (1). The presence of horizontal pleiotropy may violate the second and third assumption and bias causal effect estimate. The MR-Egger method, weighted median method, and leave-one-out analysis were operated to measure the horizontal pleiotropy. MR-Egger method combines Wald ratios into a meta-regression (with an intercept and slope parameter) to estimate the causal effect adjusted for any horizontal pleiotropy. (as demonstrated in <https://mr-dictionary.mrcieu.ac.uk/term/mr-egger/>). MR-Egger is an analysis method for MR using summarized genetic data, which consists of three parts: (i) a test for horizontal pleiotropy, (ii) a test for a causal effect, and (iii) an estimate of the causal effect(3). While conventional analysis methods for Mendelian randomization assume that all genetic variants satisfy the instrumental variable assumptions, the MR-Egger method is able to assess whether genetic variants have pleiotropic effects on the outcome that differ on average from zero (horizontal pleiotropy), as well as to provide a consistent estimate of the causal effect, under a weaker assumption— the InSIDE (INstrument Strength Independent of Direct Effect) assumption(3). InSIDE assumption states that the association between the genetic instrument and exposure is not correlated with a path from the genetic instrument to the outcome independent of the exposure of interest (as demonstrated in <https://mr-dictionary.mrcieu.ac.uk/term/inside/>). The intercept of an MR-Egger regression provides an indication of pleiotropy when it is not null. MR-Egger method returns an unbiased causal effect even if the second and third assumption is violated for all SNPs but assumes that the horizontal pleiotropic effects are not correlated with the SNP-exposure effects (1). Our result of MR-Egger showed that intercept (measurement of horizontal pleiotropy) is not significantly different from 0 (p>0.05), which indicates that horizontal pleiotropy is not presented in our study. The leave-one-out analysis is also performed to evaluate if the MR estimate is driven or biased by a single SNP that might have a particularly large horizontal pleiotropic effect (1). We re-estimate the effect by sequentially dropping one SNP at a time (1). For example, we have 13 SNPs associated with GD in our study. We drop 1 SNP and use 12 left SNPs to perform MR to observe whether the result changes dramatically. This process will repeat 13 times. The results would be summarized in a plot. Identifying SNPs that, when dropped, lead to a dramatic change in the estimate can be informative about the sensitivity of the estimate to outliers(1). In our study, horizontal pleiotropy is not presented according to the leave-one-out analysis. The weighted median method takes the median effect of all available SNPs. The advantage of this method is that only half the SNPs need to be valid instruments for the causal effect estimate to be unbiased. (1)The weighted median estimate allows more robust SNPs to contribute more towards the estimate and can be obtained by weighting the contribution of each SNP by the inverse variance of its association with the outcome (1).

In MR studies, where genetic variants are used as proxy measures for an exposure trait of interest, obtaining adequate statistical power is frequently a concern due to the small amount of variation in a phenotypic trait that is typically explained by genetic variants (5). Given the experimental sample size and population parameters that summarize variance explained in the exposure trait by the instrument and the causal and observational associations between the exposure and outcome, simple power calculations for a two-stage least squares MR can be generated (5). Brion’s method provides general equations for calculating statistical power for two-stage least squares MR based on asymptotic theory and implements the equations in a web-based application (5). It is a widely used method to calculate statistical power (6-8).

**Reference:**

1. Hemani G, Zheng J, Elsworth B, Wade KH, Haberland V, Baird D, et al. The MR-Base platform supports systematic causal inference across the human phenome. *Elife* (2018) 7. Epub 2018/05/31. doi: 10.7554/eLife.34408. PubMed PMID: 29846171; PubMed Central PMCID: PMCPMC5976434.

2. Sheehan NA, Didelez V. Epidemiology, genetic epidemiology and Mendelian randomisation: more need than ever to attend to detail. *Hum Genet* (2020) 139(1):121-36. Epub 2019/05/28. doi: 10.1007/s00439-019-02027-3. PubMed PMID: 31134333; PubMed Central PMCID: PMCPMC6942032.

3. Burgess S, Thompson SG. Interpreting findings from Mendelian randomization using the MR-Egger method. *Eur J Epidemiol* (2017) 32(5):377-89. Epub 2017/05/21. doi: 10.1007/s10654-017-0255-x. PubMed PMID: 28527048; PubMed Central PMCID: PMCPMC5506233.

4. Emdin CA, Khera AV, Kathiresan S. Mendelian Randomization. *Jama* (2017) 318(19):1925-6. Epub 2017/11/23. doi: 10.1001/jama.2017.17219. PubMed PMID: 29164242.

5. Brion MJ, Shakhbazov K, Visscher PM. Calculating statistical power in Mendelian randomization studies. *Int J Epidemiol* (2013) 42(5):1497-501. Epub 2013/10/26. doi: 10.1093/ije/dyt179. PubMed PMID: 24159078; PubMed Central PMCID: PMCPMC3807619.

6. Zhu J, Niu Z, Alfredsson L, Klareskog L, Padyukov L, Jiang X. Age at menarche, age at natural menopause, and risk of rheumatoid arthritis - a Mendelian randomization study. *Arthritis Res Ther* (2021) 23(1):108. Epub 2021/04/11. doi: 10.1186/s13075-021-02495-x. PubMed PMID: 33836822; PubMed Central PMCID: PMCPMC8034136.

7. Zhou H, Zhang Y, Liu J, Yang Y, Fang W, Hong S, et al. Education and lung cancer: a Mendelian randomization study. *Int J Epidemiol* (2019) 48(3):743-50. Epub 2019/06/21. doi: 10.1093/ije/dyz121. PubMed PMID: 31219597.

8. Liu H, Zhang Y, Zhang H, Wang L, Wang T, Han Z, et al. Effect of plasma vitamin C levels on Parkinson's disease and age at onset: a Mendelian randomization study. *J Transl Med* (2021) 19(1):221. Epub 2021/05/26. doi: 10.1186/s12967-021-02892-5. PubMed PMID: 34030714; PubMed Central PMCID: PMCPMC8142636.
